# Knowledge of Palliative Care in Ecuador

**DOI:** 10.3390/ijerph18094840

**Published:** 2021-04-30

**Authors:** Paula Hidalgo-Andrade, Guido Mascialino, Diego Miño, Matías Mendoza, Anna Belén Marcillo

**Affiliations:** School of Psychology, Universidad de Las Américas, Quito EC 170124, Ecuador; guido.mascialino@udla.edu.ec (G.M.); diego.mino.vargas@udla.edu.ec (D.M.); victor.mendoza@udla.edu.ec (M.M.); anna.marcillo@udla.edu.ec (A.B.M.)

**Keywords:** palliative care, knowledge, education, Ecuador

## Abstract

Palliative care is a holistic approach to care for people with chronic, advanced, and life-threatening illnesses. It improves the quality of life of patients and their caregivers. However, there is still limited access to palliative care in many countries. Knowledge about palliative care is key to increase its availability. Thus, this article aimed to explore the knowledge of the general population about palliative care in Ecuador. The study had a descriptive cross-sectional design. Through snowball sampling, between September 2019 and January 2020, 257 people completed an anonymous online questionnaire about general and specific aspects of palliative care. Descriptive and ANOVA analyses indicate that people have many misconceptions about palliative care and how it should be provided. Gender, education, training, occupation, and experience as caregivers were related to the total level of knowledge about palliative care. Regression analyses show other variables as predictors of knowledge. This study highlights the lack of knowledge about specific issues within palliative care in the general population in Ecuador. It also shows the need to develop and implement education measures to fill these gaps and enhance access to palliative care in health systems.

## 1. Introduction

Palliative care (PC) is an active and holistic approach to care for people who suffer from severe illness and especially for those near the end of life; PC seeks to improve the quality of life of patients of all ages, their families, and their caregivers [1]. Increased life expectancy, coupled with the growing incidence of chronic diseases, has highlighted the need for this type of care to contribute to the alleviation of disease-related suffering [2]. In addition to the direct and indirect social gains that PC offers to patients and their families; there are also benefits for the health systems [3,4]. It has been shown that PCs are less expensive than other types of care [5] and their effective incorporation into the health systems can reduce health costs [6,7].

Currently, the COVID-19 pandemic has revealed with greater intensity the vital importance of PC and its lack of implementation at all levels of care [8,9]. However, there is still limited availability of specialized PC resources while the quality of end-of-life care and chronic pain management remain two of the most important public health issues worldwide [10]. Despite these shortcomings, PC has slowly gained more importance and its inclusion in health-related professions curricula has been advocated [11,12,13,14]. Likewise, research has highlighted the benefits and need of increasing knowledge and education about PC in healthcare personnel [15,16,17] as well as in caregivers and patients [18,19].

To effectively promote the development of PC, it is essential to decrease the lack of understanding towards the topic; evident barriers to the work of healthcare workers in PC [20]. Given that PC seeks the well-being of patients and their families, it is necessary to investigate how they are perceived and the knowledge that the population has about them since they are the potential beneficiaries. Thus, the purpose of this study is to explore knowledge about PC and the care required by a person with palliative needs in Ecuador, a country where PC has slowly been implemented: the first PC unit was established in 1995, in 1997, the Ecuadorian Foundation for PC was created, in 1998, PC entered the medicine curricula at *Universidad Estatal de Guayaquil* and, in 2018, the Ecuadorian Association for PC was recognized by the National Ministry of Health. Ecuador has an isolated provision of PC, unequal reach, and not well-supported PC services [10]. According to recent data, Ecuador has 78 PC teams (only one of them for pediatric PC), has a “regular” opioid distribution, and only 3,5% of people who need PC receive it [21].

## 2. Materials and Methods

In this cross-sectional descriptive quantitative study, participants completed an online anonymous questionnaire on SurveyMonkey between September 2019 and January 2020, distributed social media using snowball sampling. The link to the survey was posted on the individual and professional social media (Twitter, WhatsApp, and Facebook) of the authors, the research team, and the School of Psychology of Universidad de Las Américas. The average time to complete it was 15 min and participants did not receive any compensation. The research was approved by the Bioethics Committee of the Universidad San Francisco de Quito (IR-E55-2019-CEISH-USFQ).

### 2.1. Participants

A total of 269 people entered the survey. Of these, 4.46% were eliminated for not having completed more than 75% of the survey, making the final sample 257 people. To participate, subjects had to be 18 or older, live in Ecuador, and accept the informed consent form.

### 2.2. Instruments

A questionnaire of perceptions of PC was developed based on previous literature [15,17,18,22]. The latter was reviewed and validated by experts in PC in Ecuador to ensure adaptation to the context. The four experts were active members of the Ecuadorian PC Care Association (ASECUP, for its initials in Spanish) and included a medical doctor, a psychologist, a social worker, and a nurse. This expert advisory group supported the content validity of the scale. The instrument’s reliability was calculated and found to be good (Cronbach’s alpha = 0.79). The data analyzed in this article correspond to the following four sections:

- Sociodemographic information: It included questions about participants’ age, gender, marital status, education, and if their current occupation was related to healthcare (yes/no). These questions were presented at the beginning of the survey.
- General knowledge about PC: These nine questions (items 1 through 9) were presented in a five-point Likert scale ranging from totally disagree to totally agree and included information on general aspects of PC such as when it should be provided, palliative sedation, euthanasia, costs, PC providers (who should provide care), and family involvement.
- Beliefs on how to act when faced with caring for a person with palliative needs: This section included the following instruction: “Please imagine that you are caring for a loved one (patient) with a disease chronic or degenerative and indicate how much you agree with each statement.” The 17 statements (items 10 through 26) included general information on feeding, physical pain, asphyxia, skincare, patient’s will, daily activities, spirituality, and caregiver care.
- Previous familiarity with PC: This last section included questions in a yes/no response format on previous experience as a formal or informal caregiver, having received PC themselves, having heard of PC before taking the survey. The level of familiarity with PC was also examined by a five-point Likert scale ranging from not at all familiar to extremely familiar with PC).

Each item assessing knowledge and/or beliefs is assumed to have a correct answer, derived from expert review, which is detailed in Table 1. Those answers are tallied up and higher scores are representative of higher levels of knowledge.

### 2.3. Data Analysis

Statistical procedures were carried out using SPSS version 23 (IBM Corp., Armonk, NY, USA). Descriptive and frequency analyses were performed. In addition, ANOVAs and Pearson r were conducted to explore the univariate relationship between sociodemographic variables and level of knowledge about PC (Table 1; items 1 to 26), as well as three subdomains: respect for the patient’s wishes (items 17 to 19), caregiver care (items 24 to 26), and spirituality (items 21 to 23). Significant variables were entered into each of the four linear regressions to see their joint predictive value.

## 3. Results

Of the 257 participants who completed the survey, 89 were male (34.6%) and 168 were female (65.4%) with a mean age of 43.08 (SD = 16.16). Most participants had a college or university degree (40.1%), followed by postgraduate education (36.2%), technical training (7.8%), and high school (16%). Most of the sample was single (45.9%), followed by married (39.3%). Approximately half of the sample (51.8%) reported that their actual occupation was related to health care. Of the total sample, 109 participants (42.2%) endorsed having had experience caring for others as an informal caregiver, defined as family members or friends who provide care without remuneration and without belonging to any health institution, and 20 participants (7.8%) reported having received PC.

Most participants reported having heard of PC before taking this survey (78.6%), but 68.8% were very slightly to moderately familiar with them. Table 1 includes responses to items related to PC knowledge for all participants.

The level of education (β = 0.230, *p* < 0.001), PC training (β = −0.405, *p* < 0.001), health-related occupation (β = −0.184, *p* = 0.002), and gender (β = 0.114, *p* = 0.03) were predictors of the general PC knowledge (F(4, 225) = 33.395, *p* < 0.001, R^2^_adjusted_ = 0.361), while having been a formal caregiver (β = −0.005, *p* = 0.95), informal caregiver (β = −0.018, *p* = 0.75), and/or having had personal experiences of receiving PC (β = −0.005, *p* = 0.95) were not and thus removed from the model.

Concerning the subdomain respect for the patient’s wishes, education (β = 0.202, *p* = 0.001), PC training (β = −0.177, *p* = 007), and occupation (β = −0.190, *p* = 004) all contributed significantly to the model (*F*(3, 232) = 14.51, *p* < 0.001, R^2^_adjusted_ = 0.147), but having been a formal caregiver did not (β = 0.007, *p* = 0.92). In the case of caregiver care, only having a health-related occupation (*F*(1, 241) = 5.98, *p* = 0.015) was positively associated with the level of knowledge, therefore, no regression was performed. The model for spiritual aspects in PC was significant (*F*(1, 239) = 16.45, *p* < 0.000, R^2^_adjusted_ = 0.064), with the level of education being the only significant predictor (β = 0.254, *p* < 0.001); health-related occupation (β = −0.095, *p* = 0.16), training in PC (β = −0.134, *p* = 0.10), and being a formal (β = 0.042, *p* = 0.61) or informal caregiver (β = −0.1, *p* = 0.13) were not significant and were excluded from the model.

## 4. Discussion

The results show a substantial lack of knowledge regarding the particularities of PC. Contrary to other studies where the majority of people (71.6%) had not previously heard of PC [23], in this research, 78.8% reported having heard about them. This contrast can be explained by the characteristics of the sample, which contains many health professionals and does not represent the demographics of the country. Indeed, having a health-related occupation was a predictor of higher levels of PC knowledge and correct beliefs in three of the four models. However, having heard of PC does not guarantee its understanding or access. Thus, even though more than half of the sample had health-related occupations and had heard of PC, 68.8% indicated that they were not familiar with them.

Most participants demonstrated clarity on basic aspects of PC. However, the results of the regressions performed indicate that level of education, occupation (health-related or not), having had specific training in PC, and gender were predictive of the level of general knowledge about PC. Furthermore, contrary to the very definition of PC [1], as in previous studies [24], participants report that PC should be given only when there are no other therapeutic options and that PC is equivalent to pain management. Likewise, as in other research [25], misperceptions were identified regarding where and how PC should be provided. Most participants are unaware that PC could be applied throughout the illness by professionals with basic training and at various levels of care, including at home [1]. These errors may still be contributing to one of the greatest barriers perceived by PC professionals in Ecuador and elsewhere in the world: late referral to PC that complicates symptom management and weakens support for family members [20].

Even when lack of knowledge is evident in several aspects of the generalities of PC, it is worth highlighting that most people recognize that the patient’s wishes must be respected, as well as maintaining their independence in daily activities. Once again, however, the level of education, occupation, and training in PC were predictors of this knowledge. Likewise, regarding spiritual care, the majority agreed to discuss death if the person so wishes. However, the level of education was the only predictor of better knowledge in this area. Our results indicate that the aspects included in the spiritual dimension (conversations about hope and death) may be the most neglected, perhaps because there are no specific and disseminated methodologies to address it [26]. The variability of responses in the item about providing hope to a person with a chronic, advanced, or incurable disease may indicate the still ingrained concept of “there is nothing to do” when PC emphasizes curing when possible, but always caring and alleviating suffering. Finally, most people are clear about the importance of caring for the caregiver. However, having a health-related occupation was the only significantly related variable to this area of knowledge. Interestingly, aspects such as having been a caregiver or having received PC were not. These results match those of another study where family caregivers in the USA were not familiar with PC and had many misconceptions about it, despite their active role in decision making and direct care [27]. We agree with the authors that more efforts need to be put in place to clarify the role of PC among informal caregivers.

This is the first study that shows specific knowledge about PC in Ecuador. We hope that the results will be used to address the found misconceptions and contribute to the expansion of PC and its access. However, the generalization of results should be handled with caution and some limitations should be considered. First, the data used for this study was obtained from an online survey distributed on social media, sources of information were self-reported, and there were no methods to ensure that people only answered the questionnaire once. This affected the representativeness of the sample. Moreover, being a cross-sectional descriptive study, causal interpretations cannot be made. Future studies would benefit from other recruitment methods to obtain a more representative sample, including reaching respondents from places where internet access is not available. Additionally, given that the purpose of the study was to explore the general knowledge on PC, the instrument only addressed the generalities of PC and was constructed specifically for this study, based on previous research. Future research could include more specific questions on each aspect of PC to better understand all its dimensions (biological, psychological, social, and spiritual).

## 5. Conclusions

Similar to research with the general population in another context such as the USA [28,29], this study shows that knowledge about PC in Ecuador is generally low, which may lead to under-utilization and missed opportunities to improve the quality of care for people who may benefit from it. The results also highlight some specific areas where people show more misconceptions, for example, explicitly differentiate PC from pain management, that PC can be given together with curative treatments and from the onset of the disease, that it can be provided by personnel with basic PC training (depending on the level of complexity), and that PC is are not provided only in specialized health institutions. These results also evidence the need to include PC in the curricula of healthcare professionals and related careers [11,12] and try novel efficient strategies to increase PC knowledge in the general population [30]. Moreover, although it is known that education alone is not enough to implement changes [31], it can be a first step to design and apply measures aimed at filling the gaps evidenced in the population and thus enhance access to PC from the onset of the disease and not only at the end of life.

## Figures and Tables

**Table 1:** Item responses of total PC knowledge scale.

	Totally Disagree *n* (%)	Disagree *n* (%)	Neither Agree Nor Disagree *n* (%)	Agree *n* (%)	Totally Agree *n* (%)
1. PC should only be given to people for whom curative treatments are no longer effective.	43 (16.8)	68 (26.6)	34 (13.3)	78 (30.5)	33 (12.9)
2. Some patients require sedation to alleviate suffering. *	13 (5.1)	6 (2.3)	16 (6.3)	124 (48.4)	97 (37.9)
3. PC is a form of euthanasia.	107 (41.8)	101 (39.5)	36 (14.1)	10 (3.9)	2 (0.8)
4. PC increases the cost of care.	29 (11.3)	46 (18.0)	58 (22.7)	95 (37.1)	28 (10.9)
5. PC should only be provided by specialized professionals in a hospital or nursing home.	19 (7.4)	64 (24.9)	40 (15.6)	74 (28.8)	60 (23.3)
6. The family should be involved in patient care. *	11 (4.3)	7 (2.7)	16 (6.3)	88 (34.4)	134 (52.3)
7. PC should only be provided to people with a poor prognosis (less than 6 months to live).	60 (23.4)	117 (45.7)	39 (15.2)	27 (10.5)	13 (5.1)
8. PC decreases suffering and increases quality of life. *	9 (3.5)	8 (3.1)	29 (11.3)	95 (37.1)	115 (44.9)
9. Pain management is the same as PC.	40 (15.6)	88 (34.2)	54 (21.0)	61 (23.7)	14 (5.4)
10. Patients should be offered foods rich in protein and calories so that they are well nourished because “a sick person who eats does not die”.	25 (10.3)	59 (24.3)	75 (30.9)	56 (23.0)	28 (11.5)
11. Patients should be offered food until they stop swallowing; then they should be fed by a feeding tube.	20 (8.2)	44 (18.1)	40 (16.5)	112 (46.1)	27 (11.1)
12. Food should be offered in the amount and at the time the patient desires. *	16 (6.6)	69 (28.5)	41 (16.9)	74 (30.6)	42 (17.4)
13. Analgesics should only be administered when the patient complains of pain.	24 (9.9)	91 (37.4)	33 (13.6)	73 (30.0)	22 (9.1)
14. If the patient has difficulty breathing, oxygen should be administered immediately.	7 (2.9)	22 (9.1)	41 (16.9)	109 (44.9)	64 (26.3)
15. Position changes must be continuous to avoid wounds or ulcers. *	4 (1.6)	2 (0.8)	7 (2.9)	99 (40.7)	131 (53.9)
16. The patient should remain as still as possible to avoid increasing pain or fatigue.	48 (19.8)	104 (42.8)	61 (25.1)	21 (8.6)	9 (3.7)
17. Providing detailed information about the disease should be avoided so as not to affect the patient’s emotional state.	72 (29.6)	76 (31.3)	49 (20.2)	39 (16.0)	7 (2.9)
18. The patient’s wishes regarding treatments, places of care, visits to be received, etc., must be respected. *	9 (3.7)	14 (5.8)	38 (15.6)	84 (34.6)	98 (40.3)
19. The patient should be prevented from saying goodbye to family and friends to avoid suffering.	117 (48.1)	94 (38.7)	21 (8.6)	7 (2.9)	4 (1.6)
20. If the patient’s condition permits, the patient should perform his or her daily tasks normally. *	3 (1.2)	5 (2.1)	11 (4.5)	132 (54.5)	91 (37.6)
21. Hope about life expectancy should not be given to a patient who has a chronic, advanced, and/or incurable disease.	35 (14.4)	52 (21.4)	67 (27.6)	68 (28.0)	21 (8.6)
22. If the patient wishes, death should be discussed openly with him and his next of kin. *	2 (0.8)	4 (1.7)	13 (5.4)	115 (47.5)	108 (44.6)
23. Conversations about death should be avoided because they may be an invitation to its arrival.	104 (43.0)	82 (33.9)	31 (12.8)	21 (8.7)	4 (1.7)
24. At this critical time for my patient, I must give him my full attention.	5 (2.1)	7 (2.9)	27 (11.1)	102 (42.0)	102 (42.0)
25. As a caregiver I must allow myself time for leisure, recreation, and humor. *	8 (3.3)	6 (2.5)	14 (5.8)	107 (44.0)	108 (44.4)
26. Emotional support to caregivers should be given only when they present symptoms or signs of depression or anxiety.	93 (38.3)	101 (41.6)	20 (8.2)	20 (8.2)	9 (3.7)

Note: valid percent is reported. Asterisks (*) are placed next to items for which agreement is the correct response, while the absence of it indicates disagreement is the correct response.

## Data Availability

The data presented in this study are available on reasonable request from the corresponding author. The data are not publicly available due to ongoing analysis.
